# The effects of dashboard design form on driving information reading performance under different time pressures

**DOI:** 10.3389/fpsyg.2025.1635951

**Published:** 2025-10-30

**Authors:** Yunxing Liu, Zhangfan Shen

**Affiliations:** ^1^Department of Industrial Design, Eindhoven University of Technology, Eindhoven, Netherlands; ^2^School of Design, Jiangnan University, Wuxi, China

**Keywords:** driving, dashboard, design, graphic form, data reading

## Abstract

In scenarios where driving decisions must be made rapidly, an optimally designed dashboard is crucial for maintaining driver concentration and facilitating precise decision-making. This study examines the influence of four principal aspects of dashboard design—graphical form, scale precision, indicator type, and external time pressure—on users' performance in reading information. Thirty postgraduate students engaged in a visual information recognition task comprising 288 trials, during which reaction time (RT) and absolute error (AE) were documented as dependent variables. Results showed that under both stringent (2,000 ms) and moderate (4,000 ms) time constraints, dashboards incorporating a semicircular or horizontal bar design, along with a progress-bar-type indicator and low scale precision (10), resulted in significantly faster RT and reduced AE. Conversely, circular dashboards generally exhibited subpar performance, particularly under increased time pressure. Additionally, a notable interaction between graphical form and time pressure was observed, indicating that circular dashboards were particularly susceptible when time was restricted. These findings provide practical guidance for designing driver-machine interfaces in safety-critical environments and concurrently contribute to theoretical developments in visual perception, human-computer interaction, and applied design methodologies.

## 1 Introduction

Data visualization is an effective way to visually present complex data using visual elements such as graphs and charts. It is widely applied in automotive driving control, ship traffic, aircraft navigation display, factory equipment monitoring, and many other fields (Ziakopoulos et al., 2019; Huang et al., 2020; Ohneiser et al., 2019; Baldauf et al., 2021). As an important tool for information gathering and decision-making, data visualization helps users better understand data intuitively and significantly reduces cognitive load (Faiola et al., 2015). However, an unreasonable visual form of data presentation may cause users to miss or misread important data, leading to incorrect judgments. For example, research indicates that additional information while driving may distract drivers or reduce their attentiveness (e.g., Regan et al., 2011). Especially when users have low graphical literacy or when designs are ineffective, it is difficult for many drivers to interpret complex information (Franconeri et al., 2021), which has become one of the main causes of accidents. Therefore, in complex driving scenarios, it is particularly crucial to design a data visualization form that not only meets user needs, but is also concise and easy to read. More recent simulator studies confirm that even brief visual distractions exceeding 2 s substantially degrade lane-keeping and elevate mental workload (Liang et al., 2024).

Data reading, as a process, entails the extraction of specific information from data sources utilizing the eyes, the brain, and the perceptual system (Fisher, 1975; Adler et al., 1998). This process encompasses the user's ability to comprehend pertinent data and trends derived from data visualizations and finds application in various fields, as cited in Börner et al. (2019). The assessment of the information visualization reference model, as proposed by Card, is recognized as one of the quintessential theoretical frameworks in visual analysis. This model posits that information visualization constitutes a flexible sequence of transformation processes, converting raw data into visual forms comprehensible to human perceptual and cognitive systems (Card et al., 1999). The concept of visual mapping is central to this model. Typically, data cannot be inherently mapped to geometric physical space. Therefore, it is necessary to artificially create visual representations and map data to graphic attribute structures that can be visually perceived to represent the meaning of the data (Card, 2009). When people recognize and read these visual graphics, they rely on human visual perception and cognitive abilities. After visual information is quickly acquired and presented through visual perception, cognitive processing occurs in the cerebral cortex, where processing is much slower than visual processing. Among them, inappropriate visual information forms can add a high cognitive load to the cognitive system. Therefore, data reading of information interfaces is considered a complex cognitive task (Steichen et al., 2013; Francese et al., 2022). Recent empirical work during take-over requests in partially automated vehicles likewise underscores how overloaded HMIs inflate subjective and objective workload metrics (Rodak et al., 2025).

Many studies have explored the impact of data visualization form on reading performance (Schonlau and Peters, 2012; Francois et al., 2019; Xiao et al., 2023). Among these factors, graphic form is one of the key elements influencing the visualization form. Different types of charts can convey different information according to the nature and purpose of the data, which will not only directly affect the readability of the data (McMonnies, 1999; Borgo et al., 2012), but also affect the user's reading experience (Wang et al., 2019; Cheng et al., 2023). Therefore, choosing a reasonable graphic style is the key to ensuring that users understand the relevant data accurately and quickly (Evergreen, 2019). Common graphic styles include column, line, scatter, pie, bar, and area graphs (Prasad et al., 2007; Bajic et al., 2019; Dadhich et al., 2021; Farahani et al., 2023). Some scholars have compared the differences in user perception between different data graphic forms. For example, Abeynayake et al. (2023) designed an experiment on the perceived ease of use and effectiveness of different graphic forms. The subjects watched four kinds of graph: bar, circular, line, radar, and bubble graph, and answered related questions. The results showed that bar and line graphs were easier to perceive and more effective than circular and radial graphs. Xiao et al. (2023) adopted a dual task paradigm to observe how the participants balance the main task and estimate the proportion of different graphic data. The results showed that vertical stacked graphs were better suited for quantitative reading under temporary distraction conditions. In addition, some scholars had also investigated the reading performance associated with various graphic forms. Francois et al. (2019) explored the impact of different forms and orientations of truck digital instruments on readings. The results proved that horizontally oriented dashboards are suitable for quantitative readings. Linear dashboard were suitable for qualitative readings, while circular were not. Similarly, Graham (1956) investigated the effect of horizontal, vertical, and circular graph on the speed and accuracy of reading data. Another modern HUD layout experiment showed that a horizontally compact information cluster enables the fastest target acquisition, echoing the advantages previously attributed to horizontal gauges (Li et al., 2024). Their findings indicated that the reading efficiency of horizontal direction graphs was higher than that of vertical direction graphs. This was because the width of the field of view was greater than the height. The eyes scan faster in the horizontal direction, and the accuracy would be higher. The above studies show that appropriate graphic forms can reduce users' cognitive load in the data analysis process and play an important role in improving reading performance.

However, in real driving situations, drivers rarely have enough time to focus on reading data on the interactive screen. NHTSA Guidelines mention that taking the driver's eyes away from the road ahead for more than 2 s will significantly increase driving risks: for engaging in activities that require proper concentration occupied in vehicle accidents caused by distraction was higher to 14–21% (NHTSA, 2013). When time-critical take-over requests occur, an information-assisted modality that fuses lane and hazard cues markedly shortens decision latency (Yu et al., 2025). Obviously, although the studies mentioned above extensively covered the reading of different graphic forms, they overlooked the potential impact of time pressure on data interpretation. Will relevant research results change under certain time pressure? It seems to still be unknown. In addition, most of the previous research on visualization form has ignored two important design elements: indicators and scales. Indicators are designed to guide users to pay attention to specific data points or progress changes. Currently, common designs for interactive screens and instruments include filling indicators, linear pointers (Li et al., 2011; Yang et al., 2022) and non-linear pointers (Li et al., 2021). Scales are used in charts to mark and quantify the size of data, providing users with the basic measurement and relative relationship of data.

This study systematically deconstructed data charts, focusing on assessing the influence of graphical form, scale precision, indicators, and external temporal pressure on reading performance. The research was advanced through the development of controlled experiments designed to replicate authentic user interactions, with the goal of identifying effective forms of data visualization under specific temporal constraints. In addition, the study investigated the potential interactions among the triad of design factors and time pressure. The objective of this investigation was to systematically analyze data visualizations and determine how graphical form, scale precision, indicators, and externally imposed time constraints collectively affected reading performance. To this end, a rigorously constructed experimental protocol was employed to simulate real-world conditions involving time-constraineds information processing. Particular emphasis was placed on how participants retrieved numerical data under varying scenarios. Furthermore, the study explored interaction effects between key design elements and temporal pressure, aiming to identify optimal visualization strategies for high-stakes environments, such as vehicular interfaces or mission-critical dashboards.

## 2 Methods

### 2.1 Participants

A total of 30 postgraduate students (15 males and 15 females, ages 22 to 25, mean = 23.1, SD = 0.8) were recruited from a Chinese university for this study. All participants had never participated in similar experiments before and had normal or corrected vision without color blindness or color weakness according to consent. Holding a driving license is a compulsory. Each participant was paid 50 to 100 Chinese Yuan based on performance.

### 2.2 Apparatus

The experiment was carried out in the ergonomics laboratory, under normal lighting (around 300 lux). The experimental device was a ROG Zephyrus M16 GU603ZM laptop with a 15.9-inch LED display, featuring a resolution of 2,560 × 1,600 and a refresh rate of 165 Hz. The experimental program used to present stimuli and collect responses was developed in Unity. The distance between the participant and the display screen was ~50 cm.

### 2.3 Experimental design

#### 2.3.1 Independent variables

The present experiment was structured to examine the effects of four independent variables. The initial variable, **time pressure**, consists of two temporal conditions: 2,000 ms and 4,000 ms. The second variable, **indicator type**, provides configurations such as a progress bar, a pointer, and a configuration that combines both a progress bar and a pointer. The third variable, **scale precision**, explores levels of precision at 10, 5, and 2 by sacle. The fourth variable, **graphic form**, entails various spatial configurations, namely a vertical bar, a horizontal bar, a circular format, and a semicircular configuration.

**Figure 1:**
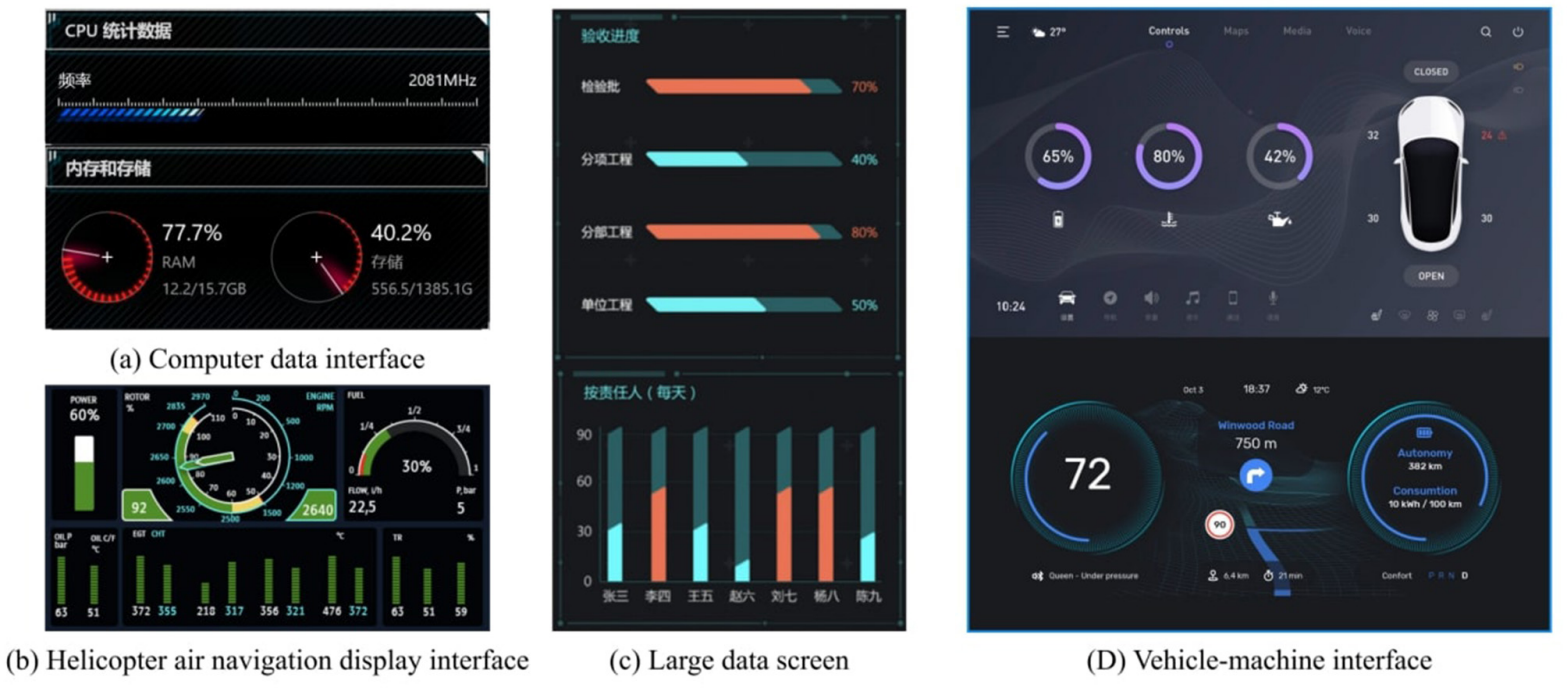
**(a–d)** Examples of data graphic forms for different fields.

The selection of the graphic form was based on analysis in multiple domains such as automobiles, aircraft, electronic devices, and data screens. Figure 1 illustrates the four commonly used graphic forms that served as levels for this independent variable.

#### 2.3.2 Indicator

**Figure 2:**
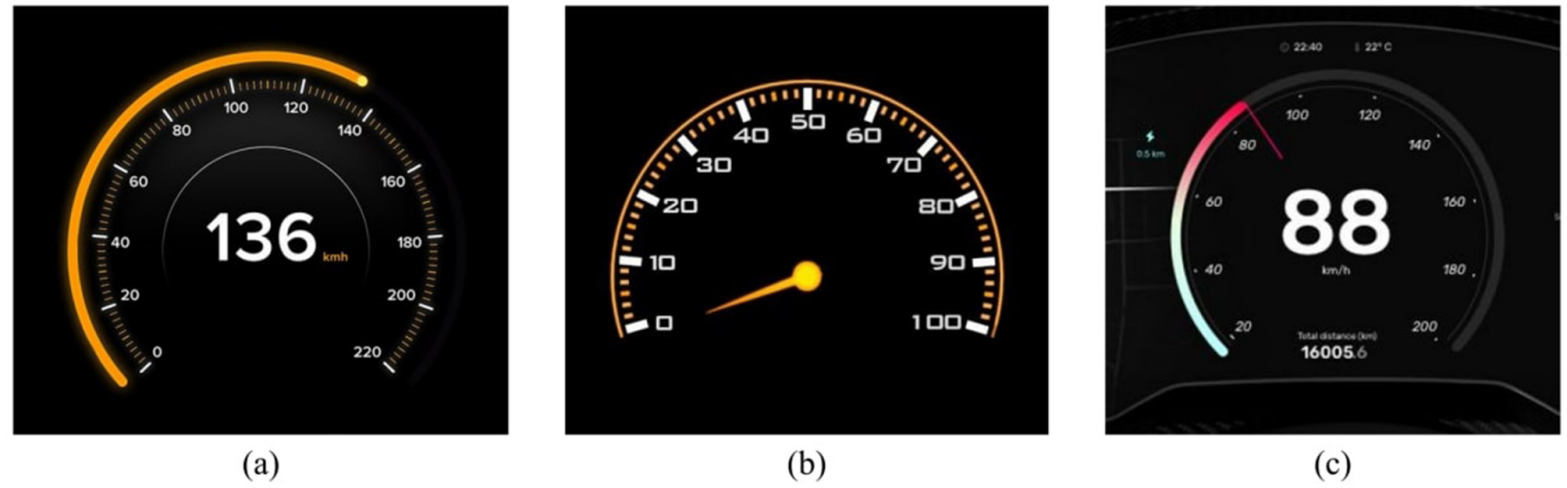
Examples of different numerical indication forms.

As shown in Figure 2, different numerical indication forms in interactive interfaces, each with advantages in specific scenarios. The progress bar displays real-time status, the dial pointer allows for rapid and precise readings, and the combination of both allows simultaneous progress and numerical representation. These were chosen as three levels for the indicator variable.

#### 2.3.3 Scale precision

**Figure 3:**
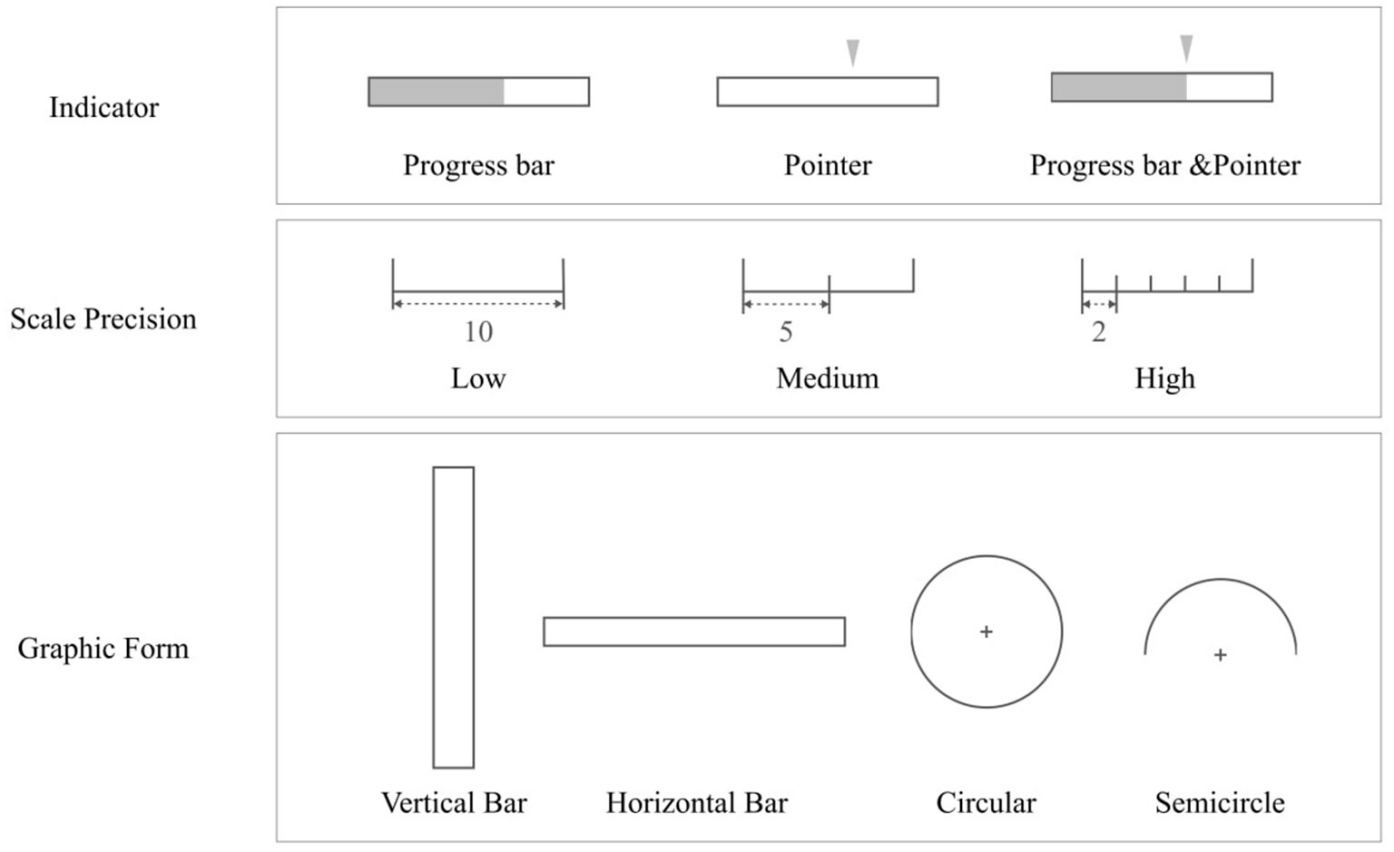
Examples of different scale precision levels.

Scale constitutes a crucial design factor that facilitates users in the interpretation of data. Conventionally, the increments of 10, 5, and 2 are employed. These three levels have been chosen to correspond to low, medium and high calibration accuracy within this study. Figure 3 demonstrates these variations.

#### 2.3.4 Time pressure

A preliminary study was conducted to determine appropriate time pressure levels. Initially, three levels (2,000 ms, 4,000 ms, and 6,000 ms) were tested with 10 volunteers. The participants interpreted various charts and input values and recorded response times. The results indicated significant effects of time pressure on the response time (*F*_2, 4419_ = 35.130, *p* < 0.001). Table 1 presents the results of the multiple comparisons of Tukey. As response times stabilized beyond 4,000 ms, the 6,000 ms level was discarded, and 2,000 ms and 4,000 ms were chosen for the formal experiment.

**Table 1:** Multiple comparisons of time pressure for RT.

**(I)**	**(J)**	**Mean difference (I–J)**	***p***	**Lower**	**Upper**
2,000	4,000	0.65503	<0.001***	0.4326	0.8774
2,000	6,000	0.71805	<0.001***	0.4955	0.9405
4,000	6,000	0.06302	0.784	−0.1595	0.2856

***p < 0.001.

#### 2.3.5 Independent variables

Two dependent variables in the experiment were reaction time (RT) and accuracy of execution (absolute error, AE). The response time (RT) refers to the time it takes for the chart page to transfer to the numerical input page and for the subject to complete their input after the fixed time of the graphic combination presentation. AE represents the absolute value of the difference between the reading and the correct value. The experimental program automatically recorded each dependent variable.

### 2.4 Materials

**Figure 4:**
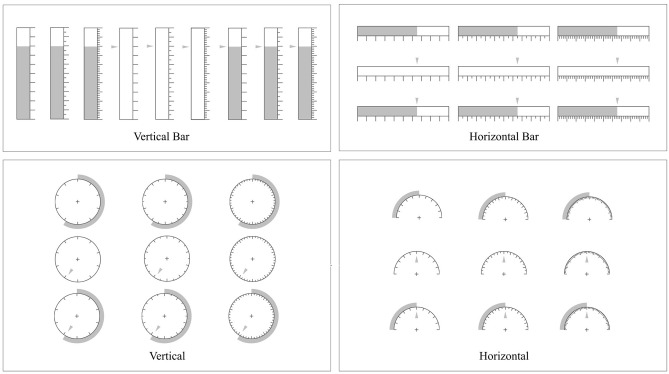
Materials of the experiment.

Figure 4 shows the different combinations of graphs used in this experiment, including 36 groups (3 numerical indication forms × 3 levels of precision × 4 types of graphs), with 9 groups in each graphic form.

### 2.5 Procedure

A visual information recognition task was designed to explore the effects of numerical indication forms, accuracy intervals, and graphic form on the data reading performance of participants under different time pressures.

According to time pressure (2,000 ms and 4,000 ms), the experiment was divided into Task 1 and Task 2, with 36 groups of chart combinations in each task. To eliminate potential distractions, strict rules were established for selecting values to be read: (a) Each combination chart was randomly generated once in the range of 0–25, 26–50, 51–75, and 76–100, totaling four times; (b) the value was not a multiple of 5 to avoid oversimplification.

Each participant completed 288 tests (2 time pressures × 3 indicator types × 3 accuracy intervals × 4 graphic forms × 4 repetitions). Participants were familiarized with different types of experiments at the beginning, and these training trials were not counted in the aphic forms and trained before the experiment, and these training trials were not counted in the formal experiment.

**Figure 5:**
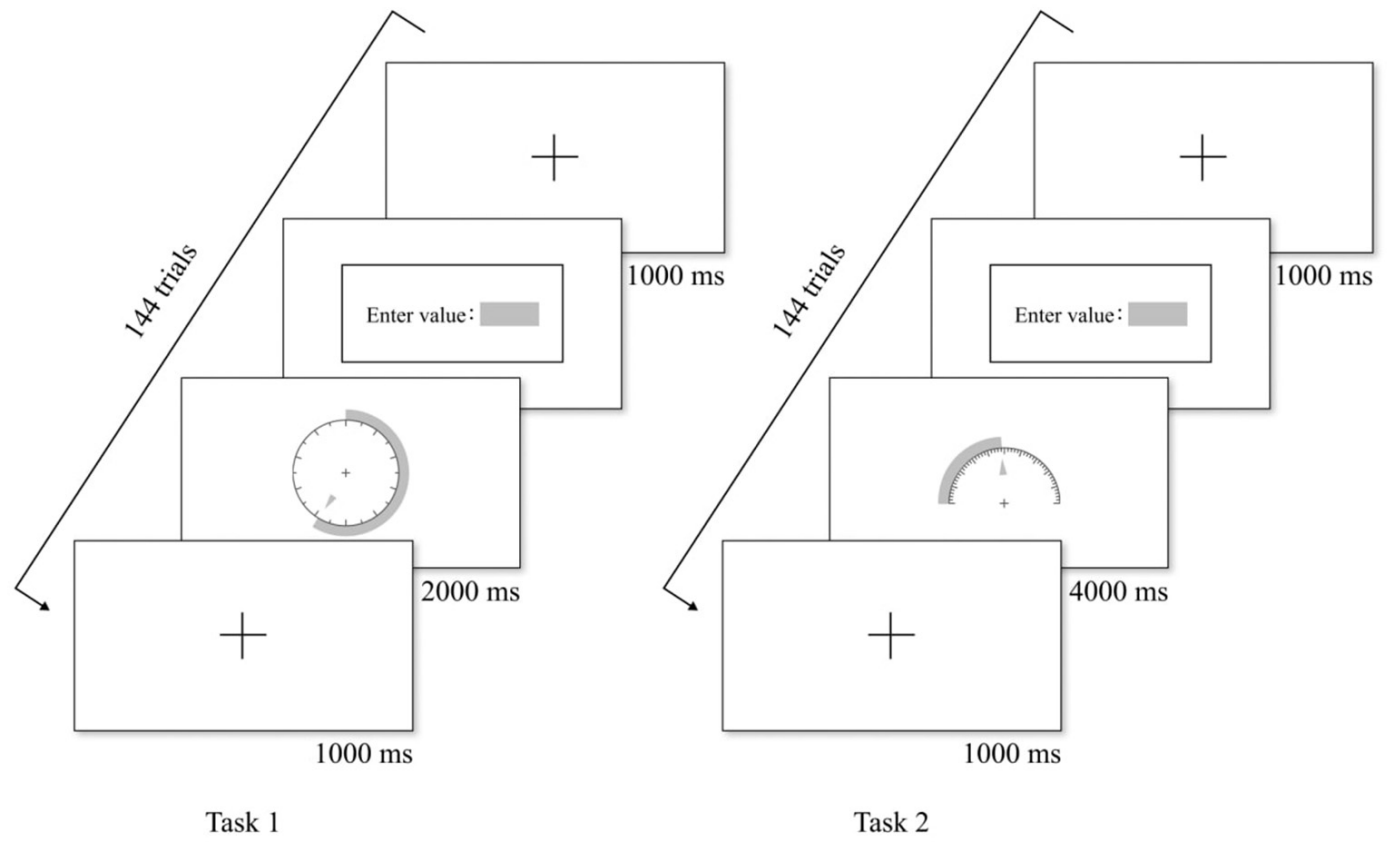
Example of experimental flow chart.

Figure 5 shows the experimental flowchart of Task 1 and Task 2. In Task 1, at the beginning of each trial, a visual focus appeared in the center of the screen for 1,000 ms. Then a combination chart appeared randomly and the participant read the numerical value. The chart was presented for a fixed time of 2,000 ms, after which the page transitioned to the input page. The participant then entered their estimated value and pressed the space bar to complete the trial. In Task 2, the only difference was the fixed presentation time, which was 4,000 ms instead of 2,000 ms.

Among the 30 participants, 15 performed Task 1 (144 trials) first, followed by Task 2 (144 trials), while the other 15 followed the reverse order. Each graphic form had nine combinations, each combination appearing four times randomly. To avoid fatigue, the participants took a 3-min break before proceeding to the next task. Each participant took ~40 min to complete both tasks.

### 2.6 Data collection and analysis

The feedback of numerical input was obtained via the keyboard. The data of the dependent variable was recorded using the designed Unity custom experimental program. Analysis of variance and *post hoc* tests were conducted using R.

## 3 Results

We recorded 8,640 trials. Following the pre-registered exclusion rules—(i) per-participant outlier removal for response time (RT) using a 3 × median absolute deviation (MAD) criterion around each participant's median and (ii) removal of absolute-error (AE) trials ≥25 (miscued key presses)—8,047 trials (93.1%) remained for analysis. All factors were within-subjects in a fully crossed 4 × 2 × 3 × 2 design: *Graphic Form* (vertical bar, horizontal bar, circular, semicircular), *Indicator* (pointer vs. progress-bar,+,pointer), *Scale Precision* (10/5/2 units per tick), and *Time Pressure* (2 s vs. 4 s). Prior to inferential testing, trial-level observations were aggregated to participant × condition cell means in Table 2.

**Table 2:** Mean and standard deviation of response time (RT) and absolute error (AE) for each level of the independent variables (*n* = 30).

**Variables**	**Number of trials**	**AE mean**	**AE SD**	**RT mean (ms)**	**RT SD**
Time pressure	8,640	1.105	2.490	2,502	2,041
2,000 ms	4,320	1.353	2.723	2,934	2,513
4,000 ms	4,320	0.855	2.203	2,067	1,274
Graphic form	8,640	1.105	2.490	2,502	2,041
Vertical bar (V)	2,160	1.178	2.420	2,604	1,999
Horizontal bar (H)	2,160	1.014	2.317	2,322	1,825
Circular (C)	2,160	1.286	2.901	2,830	2,493
Semicircle (S)	2,160	0.943	2.260	2,253	1,710
Indicator	8,640	1.105	2.490	2,502	2,041
Progress bar (PB)	2,880	1.035	2.425	2,519	2,052
Pointer (P)	2,880	1.239	2.535	2,449	2,006
Progress bar & Pointer (PB&P)	2,880	1.041	2.503	2,538	2,064
Scale precision	8,640	1.105	2.490	2,502	2,041
Low (10)	2,880	1.054	2.360	2,392	1,967
Medium (5)	2,880	1.118	2.551	2,576	2,162
High (2)	2,880	1.442	2.556	2,539	1,984

As a first step, we ran type-III repeated-measures ANOVAs on RT and AE. Because all three- and four-way interactions were far from significance in both models (all *p* > 0.50), we omit them from the summary tables for clarity and focus on main effects and two-way interactions. *F*-statistics are reported in Tables 3, 4. Where appropriate, *p*-values were Greenhouse-Geisser/Huynh-Feldt corrected for sphericity; degrees of freedom in the tables are nominal.

**Table 3:** Repeated-measures ANOVA on RT (type III).

**Effect**	**DFn**	**DFd**	**SSn**	**SSd**	***F***	***p***
(Intercept)	1	29	12,035.70	1,488.17870	234.5386575	<0.001***
TimePressure	1	29	210.2691	834.72259	7.3051868	<0.05*
Graphic Form	3	87	33.07736	75.37423	12.7264101	<0.001***
Indicator	2	58	0.793983	31.73202	0.7256237	0.4884
Precision	2	58	6.295946	26.91894	6.7826765	<0.01**
TimePressure:Graphic Form	3	87	7.198122	39.70067	5.2579855	<0.01**
TimePressure:Indicator	2	58	0.8853167	37.99068	0.6758023	0.5127
Graphic Form:Indicator	6	174	0.8371505	46.03101	0.5274132	0.7870
TimePressure:Precision	2	58	1.160825	21.39760	1.5732572	0.2161
Graphic Form:Precision	6	174	0.7378348	64.29351	0.3328051	0.9189
Indicator:Precision	4	116	1.207906	45.04311	0.7776833	0.5419

Two-way terms are reported; all three- and four-way interactions were *p*>0.50 and are omitted for brevity.

**Table 4:** Repeated-measures ANOVA on AE (type III).

**Effect**	**DFn**	**DFd**	**SSn**	**SSd**	***F***	***p***
(Intercept)	1	29	2,435.604990	360.05184	196.1732647	<0.001***
TimePressure	1	29	120.102058	119.29397	29.1964440	<0.001***
Graphic Form	3	87	27.849357	208.22530	3.8786419	<0.05*
Indicator	2	58	28.963780	63.09930	13.3115521	<0.001***
Precision	2	58	1.710847	58.97434	0.8412910	<0.01**
TimePressure:Graphic Form	3	87	3.124563	187.84978	0.4823659	<0.01**
TimePressure:Indicator	2	58	7.710693	98.32962	2.2740868	0.1120
Graphic Form:Indicator	6	174	10.730022	238.39440	1.3052766	0.2572
TimePressure:Precision	2	58	4.191287	58.30794	2.0845759	0.1336
Graphic Form:Precision	6	174	8.114127	251.41597	0.9359377	0.4706
Indicator:Precision	4	116	2.117200	205.18430	0.2992374	0.8779

Two-way terms are reported; all three- and four-way interactions were *p*>0.50 and are omitted for brevity. *p < 0.05, **p < 0.01, ***p < 0.001.

### 3.1 Time pressure

**Figure 6:**
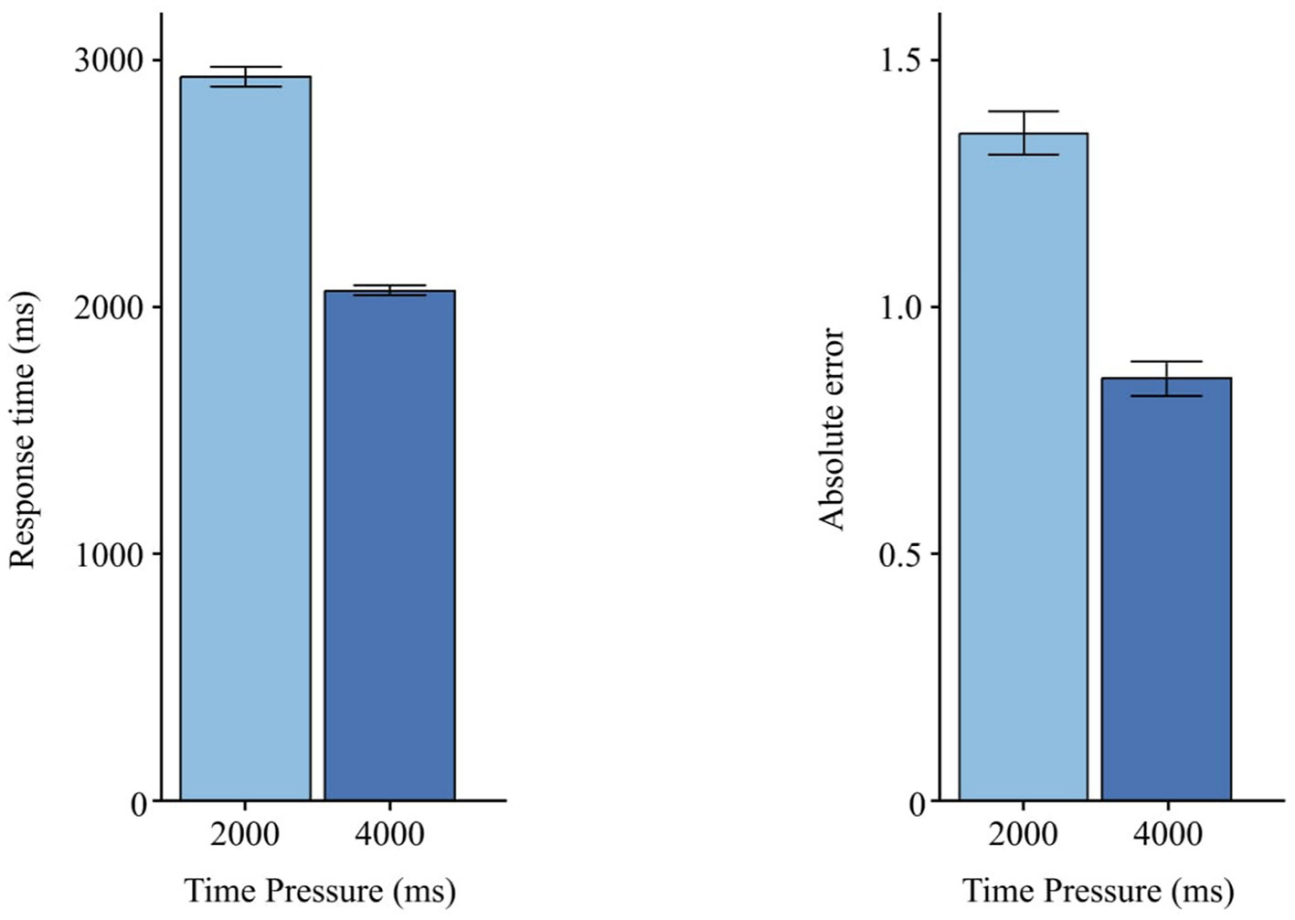
Effects of time pressure on RT and AE. Error bars indicate ±1 SE.

Across measures, a significant main effect of *Time Pressure* was observed for RT, *F*(1, 29) = 7.31, *p*= < 0.05 (Table 3), and for AE, *F*(1, 29) = 29.20, *p* < 0.001 (Table 4). Figure 6 illustrates that, compared to 2,000 ms, participants responded faster under 4,000 ms (ΔRT = 867 ms) and made fewer errors (ΔAE = 0.498).

### 3.2 Indicator

**Figure 7:**
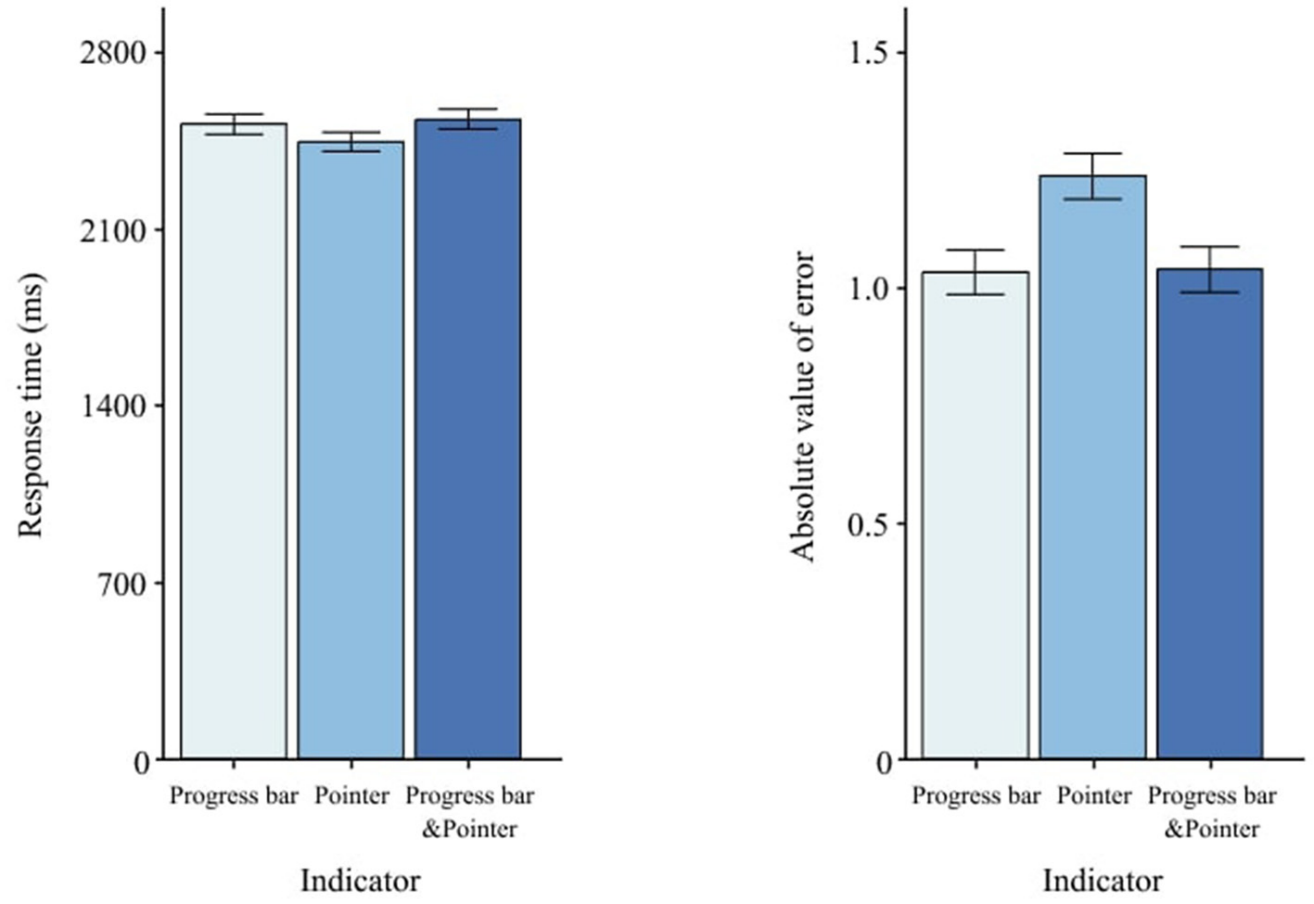
Effects of indicator type on RT and AE. Error bars indicate ±1 SE.

*Indicator* did not affect RT, *F*(2, 58) = 0.73, *p* = 0.488 (Table 3), but it significantly changed AE, *F*(2, 58) = 13.31, *p* < 0.001 (Table 4). AE for the progress bar and the combined indicator was lower than for the than for the pointer alone (Figure 7). pointer alone. *Post-hoc* Tukey pairwise comparisons (Table 5) quantify which indicator(s) reduce AE most.

**Table 5:** Multiple comparisons of indicator for AE.

**Indicator (I)**	**Indicator (J)**	**Mean difference (I–J)**	***p***	**95% Confidence interval Lower - Upper**
PB	P	−0.20324	<0.01**	−0.3636 to −0.0428
PB	PB&P	−0.00527	0.996	−0.1658 to 0.1553
P	PB&P	0.19796	<0.01**	0.0379 to 0.3579

**p < 0.01.

### 3.3 Scale precision

**Figure 8:**
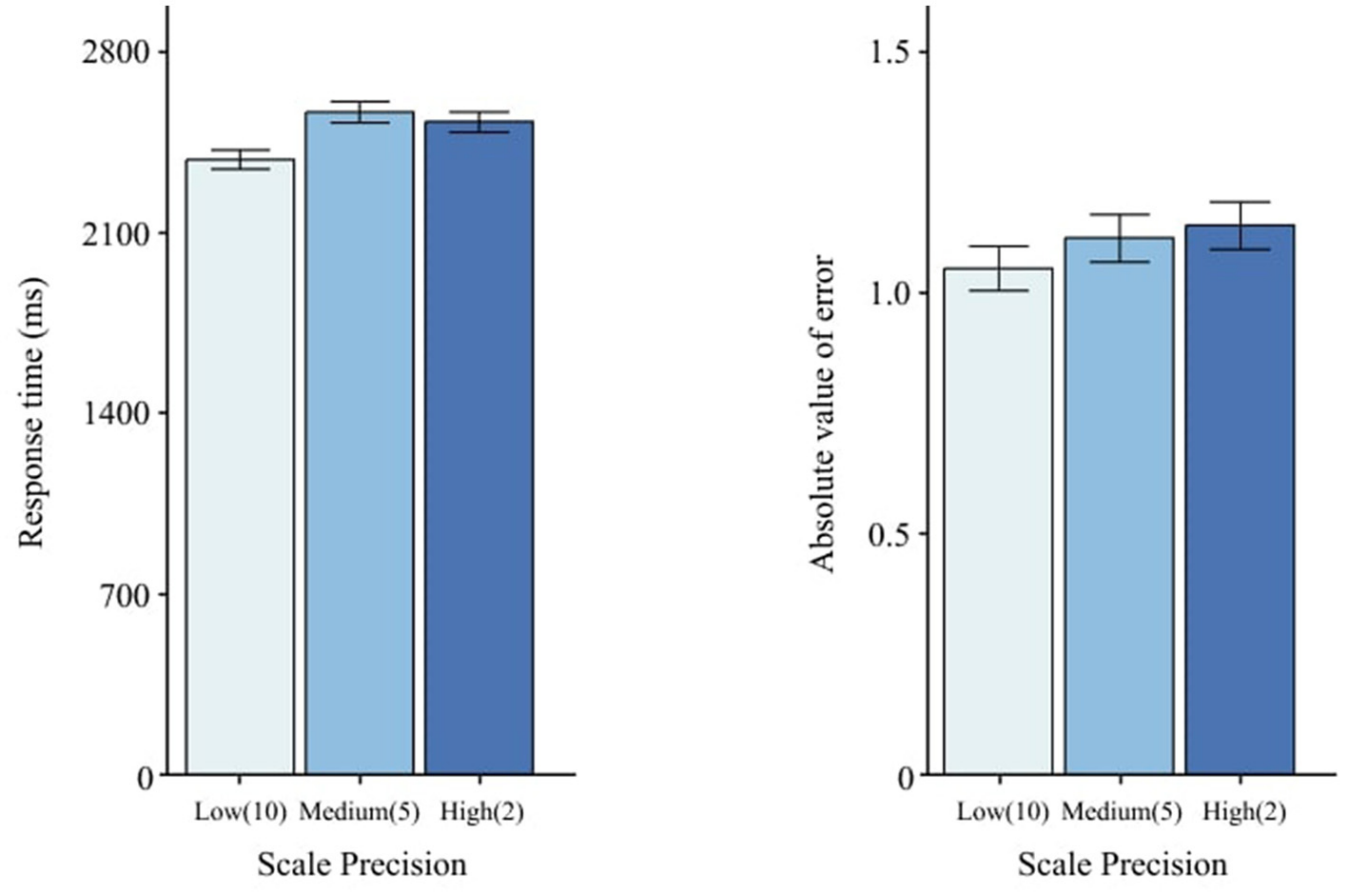
Effects of scale precision on RT and AE. Error bars indicate ±1 SE.

*Scale Precision* altered RT, *F*(2, 58) = 6.78, *p* = < 0.01 (Table 3), but not AE, *F*(2, 58) = 0.84, *p* = 0.436 (Table 4). Low precision (10) yielded faster RT than medium (5) and high (2), as shown in Figure 8.

### 3.4 Graphic form

**Figure 9:**
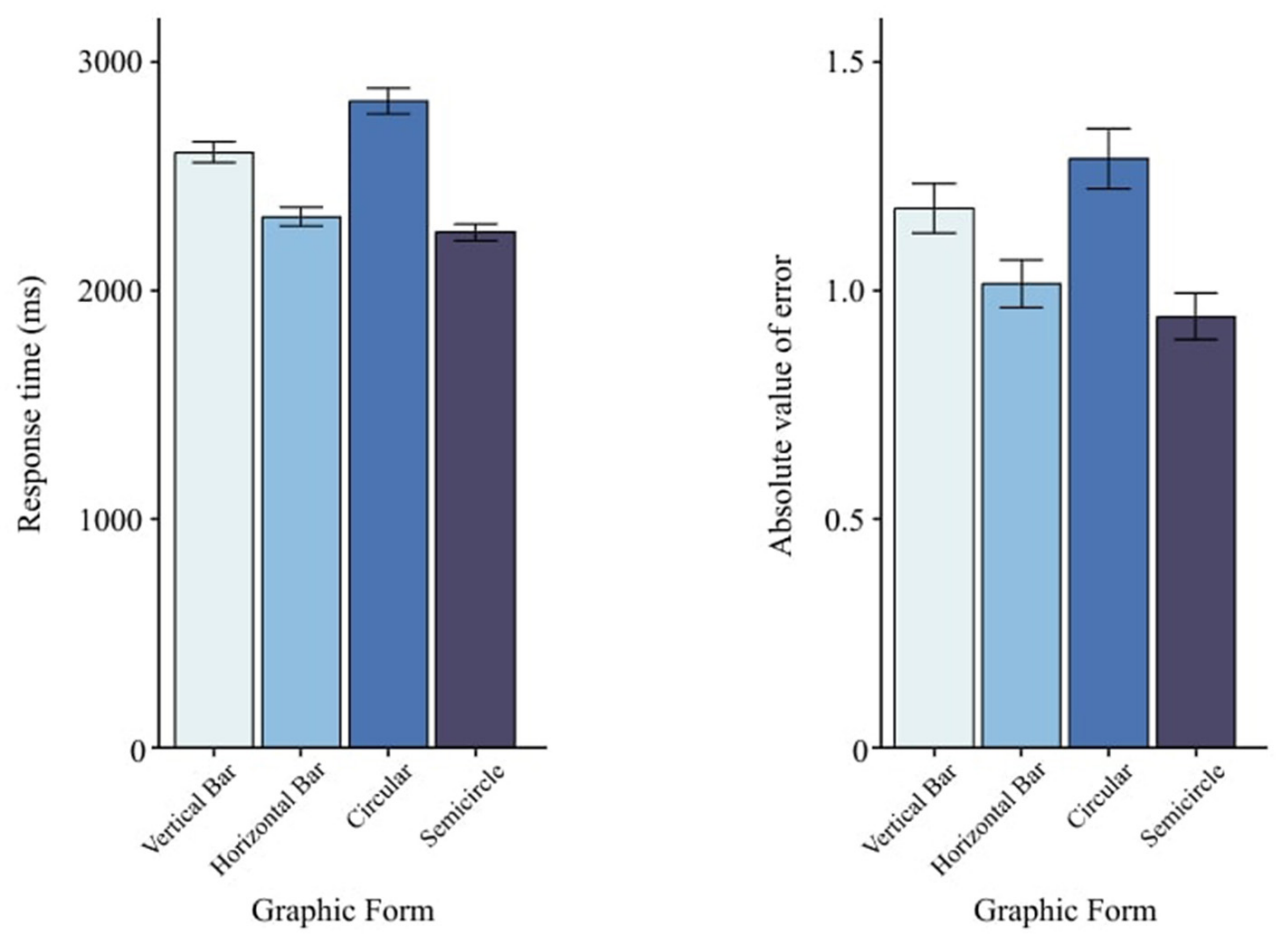
Main effects of graphic form on visual numerical information recognition task. The left panel shows RT, and the right panel shows AE. Error bars indicate ±1 SEs.

Figure 9 shows mean performance for the four *Graphic Form* levels. There were main effects of *Graphic Form* on both RT, *F*(3, 87) = 12.73, *p* < 0.001 (Table 3), and AE, *F*(3, 87) = 3.88, *p* = 0.05 (Table 4). While the omnibus tests establish that differences exist, directionality between specific pairs should be interpreted via descriptive means and planned Tukey EMM contrasts reported alongside Figure 9. Based on RT and AE, *semicircle* charts showed the best reading performance, whereas *circle* charts performed worst. Detailed Tukey results are provided in Tables 6, 7.

**Table 6:** Multiple comparisons of graphic form for RT.

**Graphic Form**		**Mean Diff (I–J)**	***p***	**Lower**	**Upper**
**(I)**	**(J)**				
V	H	281.78	<0.001***	116.73	446.83
V	C	−225.74	<0.01**	−391.13	−60.36
V	S	350.56	<0.001***	185.28	515.84
H	C	−507.53	<0.001***	−673.03	−342.02
H	S	68.78	0.708	−96.62	234.18
C	S	576.30	<0.001***	410.57	742.04

**p < 0.01; ***p < 0.001

**Table 7:** Multiple comparisons of graphic form for AE.

**Graphic Form**		**Mean Diff (I–J)**	***p***	**Lower**	**Upper**
**(I)**	**(J)**				
V	H	0.16462	0.156	−0.0377	0.3669
V	C	−0.10826	0.517	−0.3110	0.0945
V	S	0.23521	<0.05*	0.0325	0.4378
H	C	−0.27288	<0.01**	−0.4758	−0.0699
H	S	0.07059	0.807	−0.1322	0.2734
C	S	0.34348	<0.001***	0.1402	0.5466

*p < 0.05; **p < 0.01; ***p < 0.001.

### 3.5 Interaction effects

**Figure 10:**
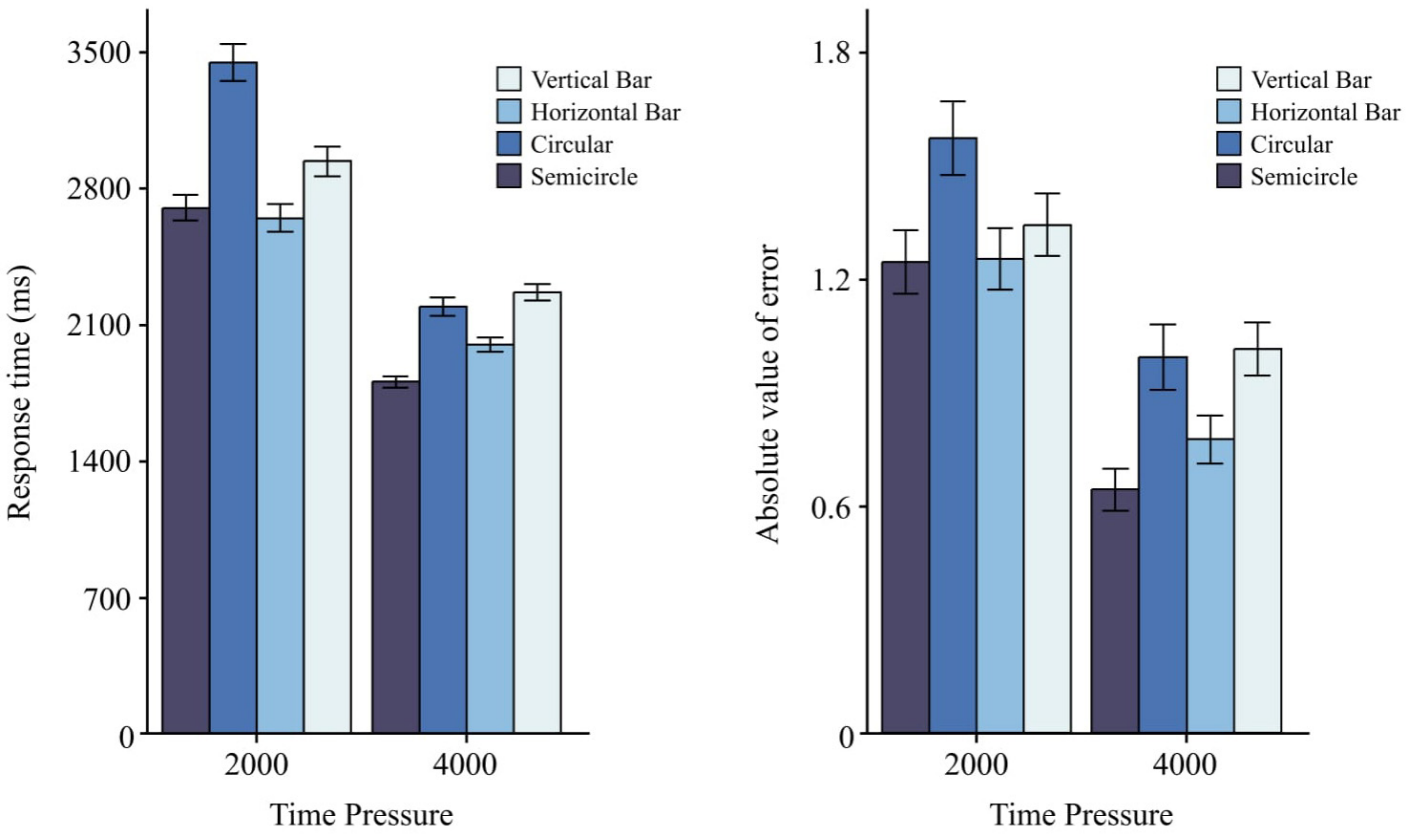
Main effects of graphic form on visual information reading task under different time pressures. The left panel shows RT, and the right panel shows AE. Error bars indicate ±1 SE.

Beyond main effects, the RM-ANOVA for RT (Table 4) revealed a *Time Pressure* × *Graphic Form* interaction, *F*(3, 87) = 5.26, < 0.01 (Figure 10). No other two-way, three-way, or four-way interactions reached significance for RT (all *p*>0.05), and *Indicator* type did not affect RT overall, *F*(2, 58) = 0.73, *p* = 0.488. For AE (Table 4), no interactions were significant (all *p*>0.05).

(Table 8) reports Tukey-adjusted pairwise contrasts for *Graphic Form* within each *Time Pressure* level (RT; positive differences indicate longer RT for the row form). Under 2s, *Circular* was reliably slower than *Horizontal, Vertical*, and *Semicircle* (all *p* < 0.001), and *Vertical* was slower than *Horizontal* (*p* = 0.021); contrasts involving *Semicircle* vs. *Horizontal*/*Vertical* were not significant. Under 4s, *Semicircle* was faster than *Vertical* (*p* < 0.001) and *Circular* (*p* < 0.001), and *Horizontal* was faster than *Vertical* (*p* < 0.05); other pairs did not differ reliably. This pattern aligns with the Repteated Measure ANOVAs (Tables 3, 4): *Graphic Form* affected both RT and AE, with *Semicircle* tending to yield the best performance and *Circular* the worst. For completeness, *Indicator* influenced AE but not RT, whereas *Scale Precision* influenced RT but not AE (Tables 3, 4).

**Table 8:** Multiple comparisons of graphic form for RT under different time pressures.

**Time pressure**	**Chart type comparison**	**Mean difference (I–J)**	***p***	**95% Confidence interval**
2 s	V vs. H	0.29268	0.021	0.0240 to 0.5613
	V vs. C	-0.50659	<0.001***	−0.7746 to -0.2385
	V vs. S	0.23882	0.125	−0.0301 to 0.5077
	H vs. C	0.79928	<0.001***	−1.0677 to -0.5308
	H vs. S	-0.05386	0.998	−0.3231 to 0.2154
	C vs. S	0.74541	<0.001***	0.4766 to 1.0141
4 s	V vs. H	0.26954	<0.05*	0.0009 to 0.5381
	V vs. C	0.07344	0.991	−0.1968 to 0.3437
	V vs. S	0.46118	<0.001***	0.1921 to 0.7302
	H vs. C	0.19610	0.352	−0.4664 to 0.0742
	H vs. S	0.19164	0.377	−0.0774 to 0.4607
	C vs. S	0.38774	<0.001***	0.1169 to 0.6584

*p < 0.05; *** *p* < 0.001.

## 4 Discussion

Previous research has primarily focused on how different graphic forms influence data-reading performance (Abeynayake et al., 2023; Xiao et al., 2023; Francois et al., 2019; Graham, 1956). These studies generally conclude that linear charts (e.g., bar or line charts) are well suited for qualitative judgments, while horizontally oriented charts often facilitate more accurate and efficient quantitative readings. However, in addition to chart type, other design elements, namely the indicator (pointer or progress bar) and scale precision, also play a critical role. Furthermore, time pressure can substantially reshape how these design factors affect performance.

### 4.1 Graphic forms

Consistent with previous work (Steichen et al., 2013; Abeynayake et al., 2023; Xiao et al., 2023), our findings confirmed that graphic forms significantly influenced reading performance. When considering both reaction time (RT) and absolute error (AE), the semicircular chart exhibited the best overall performance, followed by the horizontal bar, vertical bar, and circular charts. This aligns with the study by Francois et al. (2019), which reported that, although semicircular and linear gauges can perform similarly in objective measures, drivers subjectively favor semicircular designs.

From our post-experiment questionnaires and interviews, participants noted that the semicircular shape was visually more salient and distinct than standard horizontal or vertical bars. They also perceived it as easier to partition into quadrants when interpreting specific ranges. We attribute this advantage to the combination of angle (the arc) and length (the radial dimension) to represent data: angles support a sense of relative proportion, while radial length signals the numeric value more directly (Cleveland and McGill, 1984; Ware, 2021). Such dual encoding may boost perceptual discrimination. Additionally, semicircular charts effectively utilize both horizontal and vertical space, offering richer visual cues than charts that rely on purely horizontal or vertical formats.

By contrast, the circular chart consistently produced the lowest reading efficiency. We hypothesize two main reasons: (1) a circle often invokes the notion of a clock face, which can lead users to inadvertently map “0, 25, 50, 75” onto the familiar “0, 3, 6, 9,” creating a near-transfer interference that disrupts accuracy; and (2) humans typically find angle judgments more difficult and time-consuming, resulting in slower, less precise readings (Cleveland and McGill, 1984). Under tighter time constraints, the circular form's reliance on angle estimation likely became more susceptible to performance degradation.

### 4.2 Indicator

Our experiment also revealed that indicator design strongly affected absolute error. Both the progress-bar style and the combined pointer-plus-progress-bar style resulted in significantly lower AE compared to a stand-alone pointer. In our setup, the single-pointer design did not align fully with the scale, leaving a small gap that users had to visually “extrapolate,” whereas the progress bar design precisely connected the filled region with its corresponding tick on the scale. This alignment eliminated the need to extend one's gaze, thereby reducing perceptual error.

These findings align with the von Restorff effect (von Restorff, 1933), which states that visually salient or unusual elements attract greater attention. Because the progress bar covers a larger filled area than a thin pointer, it provides a stronger cue. Notably, the combined progress bar + pointer did not significantly outperform the progress-bar-only style, suggesting that once the bar is present, adding a pointer does not yield further accuracy gains.

Regarding reaction time, no statistically significant differences emerged between the three types of indicators. Users tended to fixate on the ultimate tick mark where the indicator terminated, a process that may be similarly fast whether the cue was a pointer or a filled progress region. Future eye-tracking studies could offer deeper insights into how these different indicator shapes guide visual search and dwell times.

### 4.3 Scale precision

Our data indicated that increasing the scale precision (from 10 to 5 or 2) significantly prolonged RT but did not materially reduce AE. According to Cognitive Load Theory (Sweller, 2011), higher task complexity elevates intrinsic load and thus slows performance. Hick's Law (Proctor and Schneider, 2018) similarly suggests that having more or finer-grained options extends decision time.

We surmise that participants facing low-precision scales (e.g., 10) were able to approximate values more quickly by locating a coarse integer interval and then estimating the offset. In contrast, denser markings under medium- or high-precision conditions (5, 2) required extra scanning effort; under strict time limits, participants may not have leveraged the added detail for improved accuracy. As a result, AE remained largely unchanged across precision levels, yet RT significantly increased at finer scales.

### 4.4 Interactions

We introduced two time-pressure levels, 2,000 ms (which is also emphasized by NHTSA guideline (NHTSA, 2013) as the 2-s rule) and 4,000 ms [following the approach by Xiao et al. (2023)], drawing on pilot data to confirm their effectiveness. Our results showed that time pressure interacted significantly with graphic form to influence RT, mirroring previous reports (Xiao et al., 2023). However, we did not detect a significant interaction between time pressure and graphic form in terms of AE.

Crucially, as time pressure intensified, the performance gap (RT and AE) between the circular chart and the other chart types widened, implying that circular displays were especially vulnerable under rapid decision-making constraints. This susceptibility probably stemmed from the need to process both horizontal and vertical angular information. Brief interruptions during the reading process may have disrupted angle estimation, causing participants to refocus and increasing both RT and error.

Moreover, under moderate time pressure (4,000 ms), the semicircular chart outperformed the horizontal bar, but under higher pressure (2,000 ms), their performances converged. Thus, while semicircular designs showed an advantage in moderately time-constrained scenarios, they did not necessarily outpace horizontal bars in extremely tight time windows.

Finally, we found no significant interaction between indicator type and graphic form or between scale precision and graphic form. It is possible that strong time constraints overshadowed or diminished any potential secondary interactions. Future research could examine more relaxed time conditions or multitasking contexts to see whether certain indicator or scale-precision choices yield notable synergies with specific chart types.

## 5 Conclusion

This study evaluated how graphic form, indicator type, scale precision, and time pressure jointly influenced users' data-reading performance, providing insights valuable to interface designers. Under a strict time constraint of 2,000 ms, both semicircular and horizontal bar charts demonstrated favorable reading performance. However, when participants had 4,000 ms, semicircular charts consistently surpassed the other chart types, whereas circular charts were the least effective. Regarding indicator type, dashboards featuring either a progress bar alone or in combination with a pointer achieved markedly higher accuracy than those featuring only a pointer. Furthermore, employing a low scale precision (with a unit of 10) resulted in the fastest reading times, while medium or high precision (5 and 2, respectively) did not significantly enhance accuracy.

These findings hold practical relevance for designing interfaces such as real-time dashboards and driving displays, where quick and reliable data interpretation can be crucial. In contexts demanding immediate decisions, designers may wish to prioritize semicircular or horizontal charts, adopt progress-bar-style indicators, and use low-scale precision to balance speed and accuracy. Where visual space permits and time constraints are moderate, semicircular charts can offer both strong performance and user preference benefits. Future work could extend these findings by incorporating eye-tracking studies, exploring cross-cultural samples, or introducing more complex and dynamic tasks to deepen our understanding of how graphic form, indicators, and scale precision affect rapid data reading in real-world conditions.

## 6 Limitations and future work

Although this study offers useful references for visualizing data in driving interfaces, three limitations remain. Firstly, the experiment was conducted in a static laboratory context; real driving involves a much more complex sensory environment (e.g., rapid changes in ambient illumination, vehicle vibration, and motion-induced visual load). Future work should *explicitly manipulate* these environmental factors as independent variables in high-fidelity simulators or instrumented-vehicle studies to test the robustness of glance performance. Secondly, the sample consisted solely of Chinese university students; future research should broaden *sample diversity* through international recruitment and balanced demographics (including novice and older drivers). Finally, dependent measures were limited to reaction time and absolute error; extending metrics to eye tracking (e.g., fixation counts, glance durations, scanpaths, heatmaps) and multimodal workload markers, and running longitudinal protocols, would reveal learning curves and enable detection of high-load states to inform adaptive dashboard design.

## Data Availability

The original contributions presented in the study are included in the article/supplementary material, further inquiries can be directed to the corresponding author.
